# Patient and public involvement and engagement (PPIE) in Otolaryngology research: a systematic review of randomised controlled trials

**DOI:** 10.1007/s00405-025-09515-5

**Published:** 2025-07-08

**Authors:** Sirat Lodhi, Donny Kong, Stefan Linton, Emma Stapleton

**Affiliations:** Department of Otolaryngology, Manchester Royal Infirmary, Oxford Road, Manchester, M13 9WL UK

**Keywords:** Otolaryngology, Research, Patients, Stakeholder engagement, Stakeholder participation

## Abstract

**Objective:**

Patient and public involvement and engagement (PPIE) entails working with patients and the public to shape research. The Guidance for Reporting Involvement of Patients and the Public (GRIPP2) checklist aims to improve the quality and consistency of the PPIE evidence base. This study reviews PPIE in Otolaryngology randomized controlled trials (RCTs) published since introduction of the GRIPP2.

**Methods:**

This systematic review was conducted according to the PRISMA 200 guidelines and pre-registered on the PROSPERO database CRD42023448519. A robust systematic review filtered ten manuscripts into the final analysis. Study characteristics; GRIPP2 short form categories; PPIE utilisation, role, and impact data were extracted.

**Results:**

Only 10 out of 346 (3%) of Otolaryngology RCTs published since 2018 described the use of PPIE. None referred to the GRIPP2 checklist. Most studies incorporating PPIE did so for the production and approval of patient-facing material review (50%). It was not possible to determine PPIE impact on patients, or the clinical impact of PPIE in the studies which incorporated it.

**Conclusion:**

Future Otolaryngology trials should incorporate and report PPIE, which has the potential to generate benefit for all stakeholders.

## Abstract (highlights)

- Patient and Public Involvement and Engagement (PPIE) entails working with the public, including patients, to inform and shape research.
- An increased focus on PPIE in research can be partly attributed to major research funding bodies endorsing and promoting its use.
- The GRIPP2 checklist aims to improve the quality and consistency of the PPIE evidence base.
- Since its publication, only 3% of published randomized controlled trials in Otolaryngology (ten studies our of 346) described PPIE use, and none referred to the GRIPP2 checklist.
- This systematic review could not determine PPIE impact on patients, patient satisfaction, and clinical impact, as data was either lacking or the direct impact of PPIE on clinical outcomes could not be determined.

## Introduction

Patient and Public Involvement and Engagement (PPIE) entails working with patients and public stakeholders, to inform and shape research. Its use and recognition have increased over the last decade [1]. Public involvement is defined as research being carried out ‘with’ or ‘by’ members of the public, rather than ‘to’, ‘for’ or ‘about’ them [1]. Public involvement may involve membership of steering groups, the development of research materials, and undertaking interviews with research participants [1]. In contrast, public engagement refers to providing and disseminating information and knowledge about research [1]. This may involve the delivery of science festivals, raising awareness of research through social media, and disseminating research findings to research participants [1].

An increased focus on PPIE in research can be partly attributed to major research funding bodies endorsing and promoting PPIE use. For example, in the United Kingdom (UK), the National Institute for Health Research (NIHR) requires funding applications to include descriptions of how researchers have involved the public in their study, and to include plans for evaluating the impact of PPIE [1]. This approach is also adopted by major research funding bodies internationally [2–4]. The benefits of PPIE for both research design and quality, and for stakeholders, are widely recognised [5]. It is therefore unsurprising that research bodies support its use. For example, PPIE has been shown to improve study protocol design, participant recruitment and retention, and the selection of relevant outcomes [6]. Additionally, PPIE contributors perceive their involvement as impactful, and understand that their lived experience is beneficial to research [7].

A need to develop guidance to facilitate consistent reporting of PPIE has been indentified 8, 9. The lack of consistent reporting of PPIE prevents researchers from conducting high quality research to synthesis and compare data from PPIE in research [8]. This presented a barrier for researchers and the public to understand, learn from, or improve PPIE in research [10, 11]. The lack of consistent reporting prompted the development of the original Guidance for Reporting Involvement of Patients and the Public (GRIPP), which was the starting point for strengthening the quality of PPIE research [12]. This guidance was based on systematic review evidence. Since its development, it has been recognised that achieving consensus on items required for reporting PPIE in research on a broad scale is vital in producing a reporting guideline [13].

GRIPP2 was published in August 2017^14^. It is a dedicated checklist based on consensus in the international PPIE community and involved patients as collaborative research partners during its development. Both the GRIPP and GRIPP2 aim to improve the quality and consistency of the PPIE evidence base.

Despite increased efforts to promote the utilisation of PPIE within research, few studies have assessed PPIE in surgical research. Of studies which reviewed the reporting of PPIE in surgical research, PPIE reporting has been found to be low [15, 16]. These studies are outdated however, and were published prior to the introduction of the GRIPP2 checklist. Nevertheless, these findings are concerning, as PPIE has been demonstrated to be beneficial in surgical trial designs, including participant recruitment and retention [18, 17].

The importance of PPIE in Otolaryngology research is still emerging. Otolaryngology research incorporates a wide range of interventions, from major cancer treatments to interventions which aim to improve communication and quality of life. It follows therefore that PPIE could be valuable across the spectrum of conditions managed by Otolaryngologists. PPIE in audiology research and its benefits have been noted, though studies remain limited and largely descriptive [19–21]. No studies have yet assessed the PPIE in Otolaryngology research. The aim of this study was to complete the first systematic review of PPIE in recent Otolaryngology research, published since the development of the GRIPP2 checklist.

## Methods

This systematic review was performed in accordance with the Preferred Reporting Items for Systematic Reviews and Meta-Analyses (PRISMA) recommendations [22]. All researchers worked independently, with any disagreements resolved through discussion with the senior author. Studies were deemed eligible if they reported on an Otolaryngology theme, and incorporated PPIE in any part of the study design. Studies were included in the analysis if they met both of the following criteria: registered randomised controlled trial; adult or paediatric participants. Excluded studies were those which did not focus on Otolaryngology; animal or laboratory studies; not a randomised controlled trial; unregistered randomised controlled trials.

### Information sources

Electronic literature searches were performed (16th March 2023) in Pubmed, Embase via OVID, and the Cochrane Library CENTRAL database for Otolaryngology themed studies. Broad search terms were used to identify all registered Otolaryngology studies published. The search was restricted to randomised controlled trials, human participants, English-only publications, and full text availability. Date limitations (2018 to 2023) were applied, to correlate with the GRIPP2 checklist.

### Search strategy

Search terms used are outlined in Table 1. These address variations of the terms ‘otolaryngology’ and ‘registration number’ identified through a pilot search.

**Table 1:** Search terms used to complete literature searches

Database(s)	Search terms
Pubmed	1. otolaryngology [All Fields] 2. otorhinolaryngology [All Fields] 3. otology [All Fields] 4. rhinology [All Fields] 5. laryngology [All Fields] 6. otorhinolaryngologic diseases [All Fields] 7. otorhinolaryngologic surgical procedures [All Fields] 8. 1 OR 2 OR 3 OR 4 OR 5 OR 6 OR 7 9. Registration [All Fields] 10. Registration number [All Fields] 11. Register [All Fields] 12. Registered [All Fields] 13. Registry [All Fields] 14. ClinicalTrias.gov [All Fields] 15. 9 OR 10 OR 11 OR 12 OR 13 OR 14 16. 8 AND 15
Embase via OVID	1. Exp otorhinolaryngology/ 2. regist*.mp. 3. “ClinicalTrials.gov”.mp. 4. 2 OR 3 5. 1 AND 4
The Cochrane CENTRAL library	1. Otolaryngology [MeSH]

### Selection process

Duplicates were removed from the publications identified by the search strategy. Titles and abstracts were screened using the inclusion and exclusion criteria. If further information was needed to determine whether the abstracts were eligible, particularly in relation to PPIE involvement and registration status, full texts were also screened, including footnotes and acknowledgements. Trials were deemed to be unregistered if the article lacked a registration number or registration details in any section. Short-listed studies underwent full text assessment to exclude ineligible studies. Of the studies selected for full text assessment, reference lists were screened to identify relevant studies not detected by the search. Data were then extracted in accordance with the objectives and analysed.

### Data collection and summary measures

Study characteristic data were extracted for each study (Table 2). This included the year of publication and Otolaryngology theme categorised by subspecialty. Data items based on the GRIPP2 short form categories were extracted [14]. This includes whether or not studies commented on the aims, methods, results, discussion and conclusion, and reflection in relation to PPIE involvement in the study.

**Table 2:** Data items for extraction from selected studies

Data item category	Data item for extraction
GRIPP2	Inclusion or absence of aims, methods, results, discussions/conclusion, and reflection in relation to PPIE involvement in study ? mean GRIPP2 scores
RCT incorporating PPIE	Number and percentage of studies incorporating PPIE, as a proportion of the sample of papers screened in full Stage of study in which PPIE was incorporated
Outcome	PPIE impact on patients/participants as stated by authors PPIE impact on patient satisfaction, and patient satisfaction scores were included, as stated by authors PPIE impact on clinical outcomes where stated by authors Adverse events reported in studies: type and number and/or percentage of the population and/or of the type of adverse effect Whether adverse event outcomes were attributed to PPIE input and/or the intervention

The number and percentage of studies including PPIE in the sample of studies undergoing full text screening was calculated. Additional data items of interest extracted for assessment included: the role of PPIE in the study (i.e. stage of involvement), and PPIE impact on patients/study participants, patient satisfaction, and clinical outcomes. Adverse event data and whether adverse events had been attributed to the intervention and/or PPIE were noted.

Statistical analyses included calculating the total number of studies and/or the percentage of studies pertaining to each data item, where appropriate. Depending on the variable assessed and where possible, average/mean scores were also calculated (e.g. patient satisfaction scores).

### Study risk of bias assessment

Risk of bias assessments were completed using the ROB2, risk of bias version 2 (Cochrane) assessment tool [23]. The short form was used to assess five domains which addressed the risk of bias arising from the randomisation process, deviation from intended interventions, missing outcome data, measurement of the outcome, and selection of the reported result. An overall risk of bias judgement was calculated (low, high, some concerns).

## Results

**Fig. 1:**
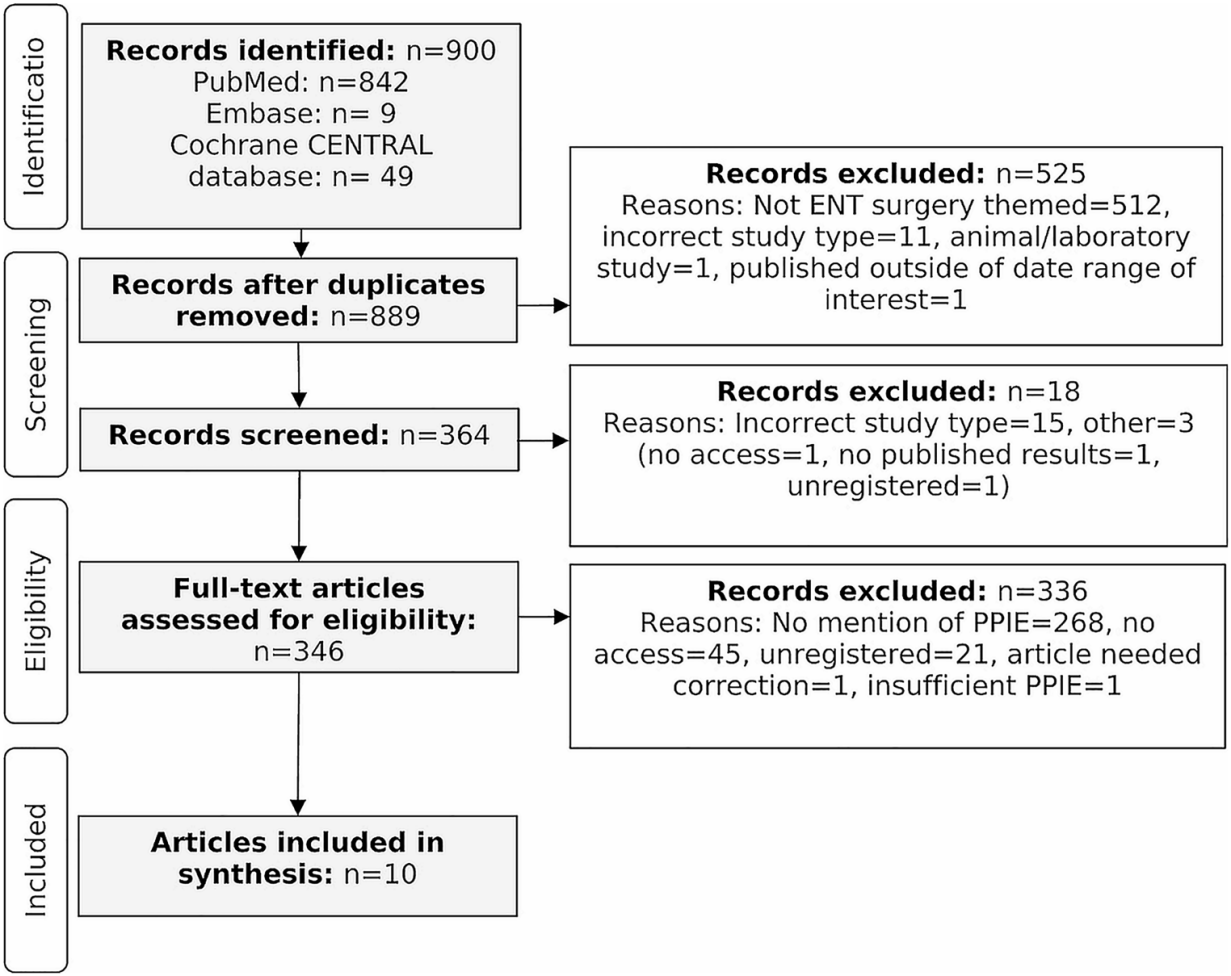
Flow diagram displaying the systematic search methodology

A four-phase PRISMA flow diagram displays the literature search (Fig. 1). Initially, 900 records were identified. After the exclusion of records based on eligibility criteria and duplication, 364 abstracts were screened. Following this, 346 articles were assessed for eligibility. This resulted in ten articles being included in the final synthesis [24–33].

### Study characteristics

The characteristics of each study, including the first author, year of publication, trial registration identification, intervention PPIE details and outcomes, and theme categorized by subspecialty, are outlined in Table 3. Four otology, two audiology, two general Otolaryngology, one rhinology, and one head and neck-themed studies were identified.

**Table 3:** Characteristics of studies included in the final synthesis

Author	Year	Trial registry ID	Theme	Intervention	PPIE details and outcomes
Almufarrij [24]	2023	Not given	Audiology	Initial vs. real ear measurement for hearing aid fitting	PPIE input to study design and patient information sheet
Beukes [25]	2022	NCT04004260	Otology	CBT for tinnitus vs. control group	Recruitment of two individuals to pilot intervention and advise on study process, recruitment and engagement
Bosel [26]	2022	NCT02377167	Head and neck	Early tracheostomy vs. standard tracheostomy	PPIE input to outcome measurement though structured conversations with patients and their families
Carrie [27]	2023	ISRCTN16168569	Rhinology	Septoplasty vs. medical management of nasal blockage	PPIE input to study design and patient facing documents
Hay [28]	2021	ISRCTN12873692	General ENT	Immediate oral vs. immediate topical vs. delayed oral antibiotics in acute otitis media	PPIE input to protocol development and patient facing materials
Van Vugt [29]	2019	NTR5712	Otology	Internet based vestibular rehabilitation with and without physiotherapy support	PPIE input to online content usability and Dutch translation
Van Vugt [30]	2020	NTR5712	Otology	Cost effectiveness of above trial (29)	PPIE input to online content usability and Dutch translation
Van Vugt [31]	2020	NTR5712	Otology	Secondary analysis of trial (29)	PPIE input to online content usability and Dutch translation
Watson [32]	2021	ISRCTN28090877	Audiology	Hearing aid vs. hearing aid plus education programme	PPIE input to research question formation
Wilson [33]	2023	ISRCN55284102	General ENT	Tonsillectomy vs. conservative management of recurrent acute tonsillitis	PPIE input to trial design and patient facing documents

### Risk of bias assessment

The risk of bias assessment for each study is outlined in Table 4. All studies rate ‘high’ for overall risk of bias, except for a study by Almufarrij et al. (low risk) [24]. Domain 1 (randomisation) assessment identified that most studies were open-label studies, studies unable to blind due to the nature of interventions, or lack of complete blinding. Domain 2 (deviation from intended assessments) assessment identified that except for the ‘low risk’ of bias studies by Almufarrij et al. [24]and Hay et al. [28]all studies rate as having ‘some concerns’ for bias. The latter did not blind participants/researchers in full. In addition, most studies did not provide information regarding deviation from intended assessments. Domain 3 (bias as a result of missing data) analyses identified that three studies rated as having ‘high’ or ‘some concerns’ for bias in this category. These studies did not have sufficient outcome data available for nearly all participants, or did not provide sufficient information to assess this. In addition, missingness in outcome data was deemed as being dependent on its true value. Most studies with ‘high risk’ of bias in domain 4 (measurement of outcomes) did not blind outcome assessors to interventions received by patients. Domain 5 identified that all completed studies were not at risk of bias due to selective reporting of results as all predefined outcomes were reported. Risk of bias assessment results for studies included in the final analysis.

**Table 4:** Risk of bias assessment results for studies included in the final analysis

Study first author	ROB2 Version 2 domain category					
	Domain 1: Risk of bias arising from the randomisation process	Domain 2: Risk of bias due to deviations from the intended interventions (effect of assignment to intervention)	Domain 3: Risk of bias due to missing outcome data	Domain 4: Risk of bias in measurement of the outcome	Domain 5: Risk of bias in selection of the reported result	Overall risk-of-bias judgement
Almufarrij I [24]	Low	low	Low	Low concerns	low	Low
Beukes E [25]	High	some concerns	High	high risk	low	high
Bösel J [26]	High	some concerns	Low	low risk	low	high
Carrie S [27]	High	some concerns	Low	high risk	low	high
Hay AD [28]	High	low	NA - study abandoned	low risk	NA - trial abandoned	high
van Vugt VA [29]	High	some concerns	Low	low risk	low	high
van Vugt VA [30]	High	some concerns	Some concerns	low risk	low	high
van Vugt VA [31]	High	some concerns	Low	low risk	low	high
Watson J [32]	High	some concerns	High	high risk	low	high
Wilson JA [33]	High	some concerns	Low	high risk	low	high

### Proportion of randomised controlled trials incorporating PPIE

Of 346 articles assessed for eligibility through full text assessment, a small proportion of articles focused on either unregistered trials (*n* = 21), or the article required a correction (*n* = 1). Of 324 articles with confirmed or potential PPIE use, ten articles described PPIE use (3%). The remaining articles had no mention of PPIE (82.7%; *n* = 268), no access (*n* = 45), and insufficient PPIE described (0.3%; *n* = 1).

### GRIPP2

GRIPP2 assessment results are summarised in Table 5.

**Table 5:** GRIPP2 short form assessment results for studies included in the final analysis

Study first author	GRIPP2 Short form category assessment result				
	Aim	Method	Results	Discussion and conclusion	Reflection/ critical perspective
Almufarrij I [24]	Yes	Partial	Partial	Partial	No
Beukes E [25]	Yes	Partial	No	No	No
Bösel J [26]	Partial	Partial	Partial	Partial	No
Carrie S [27]	Yes	Yes	Partial	Partial	No
Hay AD [28]	Yes	Partial	Partial	Partial	No
Watson J [32]	Yes	Partial	Partial	Partial	No
Wilson JA [33]	Partial	Partial	No	No	No
van Vugt VA [29]	Yes	Partial	Partial	Partial	No
van Vugt VA [30]	Yes	Partial	Partial	Partial	No
van Vugt VA [31]	Yes	Partial	Partial	Partial	No

### GRIPP2: aims

Of the ten studies included in the final synthesis, 80% stated the aim of PPIE in the study. The remainder partially stated aims.

### GRIPP2: methods

Of the ten studies included in the final synthesis, 90% partially provided a clear description of the methods used for PPIE in the study. The majority did not provide details regarding the number of individuals forming the PPIE group. Additionally, stages incorporating PPIE involvement and actions of PPIE groups were not described in full. One study addressed PPIE methods in full.

### GRIPP2: study results

Of the ten studies included in the final synthesis, 80% partially reported the results of PPIE in the study. Both positive and negative outcomes were not reported in this group. The remaining 20% of studies did not report the results.

### GRIPP2: discussion and conclusion

Of the ten studies included in the final synthesis, 80% partially commented on the extent to which PPIE influenced the study overall. Both positive and negative effects were not discussed in this group. The remaining 20% of studies did not comment on the influence of PPIE on the study overall.

### GRIPP2: reflection/critical perspective

None of the studies included in the final synthesis critically commented or reflected on the things that went well and those that did not as a result of PPIE involvement.

### Role of PPIE

Of the ten studies included in the final synthesis, 50% stated PPIE involvement in patient facing material review, and 40% in trial design and delivery (Table 6). Studies also stated PPIE involvement in intervention development (40%), outcome measure refinement (30%), understanding barriers to and boosting recruitment (20%), and designing the research question (10%).

**Table 6:** Stage of study incorporating PPIE

Study first author	Study stages incorporating PPIE					
	Design and delivery	Designing the research question	Intervention development	Patient facing material	Outcome measure	Understanding barriers to recruitment/ boosting recruitment
Almufarrij I [24]				X		
Beukes E [25]	X		X			X
Bösel J [26]					X	
Carrie S [27]	X			X	X	
Hay AD [28]	X			X	X	
van Vugt VA [29]			X			
van Vugt VA [30]			X			
van Vugt VA [31]			X			
Watson J [32]	X	X		X		X
Wilson JA [33]				X		

### PPIE impact on patients, clinical outcomes and adverse events

It was not possible to determine PPIE impact on patients and patient satisfaction as no data were provided on this theme, in studies included in final synthesis. It was therefore not possible to determine PPIE impact on clinical outcomes, especially as intervention engagement and attrition varied between studies. Of the studies included in the final synthesis, 70% reported on whether adverse events were noted in the study (Table 7). None specifically reported whether PPIE impacted adverse event findings.

**Table 7:** Reporting of adverse events

Study first author	Reporting of adverse events in studies analysed			
	Adverse effects presence/absence stated clearly	Adverse events present	Adverse events attributed to intervention	Adverse events attributed/influenced by PPIE
Almufarrij I [24]	No	Not applicable	Not applicable	No information
Beukes E [25]	Yes	Yes	Yes	No information
Bösel J [26]	Yes	Yes	Yes	No information
Carrie S [27]	Yes	Yes	Yes	No information
Hay AD [28]	Yes	Yes	No information	No information
van Vugt VA [29]	Yes	No	Not applicable	No information
van Vugt VA [30]	No	Not applicable	Not applicable	No information
van Vugt VA [31]	No	Not applicable	Not applicable	No information
Watson J [32]	Yes	Not applicable	Not applicable	No information
Wilson JA [33]	Yes	Yes	Yes	No information

## Discussion

The aim of this systematic review was to identify and analyse the use of PPIE in Otolaryngology randomised controlled trials published since the development of the GRIPP2 checklist, and its impact. Due to poor quality reporting of PPIE, the impact of PPIE on participants, clinical outcomes, and adverse events could not be measured.

In this review, a description of PPIE utilisation was found in ten studies. The majority involved patients in the design of patient facing material (50%). Patients were least likely to be involved in design of the research question (10%). This is in line with existing literature which has identified that PPIE is most often used for the purposes of reviewing patient facing material such as information sheets, an approach often regarded as tokenistic [34]. To improve the potential for positive impact of PPIE, it is generally accepted that PPIE contributors should be engaged in the early stages of trial design [34].

In line with existing literature, this review has identified that few publications report PPIE [14, 15]. This has especially been noted in the field of surgical research [14]. For example, only 25% of NIHR funded surgical trials conducted between 2006 and 2010 detailed PPIE in grant applications, in comparison to other clinical trials [14].

Nevertheless, the absence of reporting does not necessarily equate to a lack of PPIE involvement. For example, in 2019, a survey of authors of active surgical trails in the UK found that 91.5% of active trials did incorporate PPIE [34]. Additionally, lack of reporting of planned PPIE activities, and inconsistencies between planned and reported activities in research trials are recognise [35]. In this systematic review, 268 of 346 articles which underwent full text assessment did not include details regarding the presence or absence of PPIE in the study. Thus, our estimation of only ten Otolaryngology trials incorporating PPIE is likely an underestimation.

There are several potential implications of under-reporting of PPIE, both ethical and practical. Lack of transparency about the nature of PPIE in research can make it imporrible to assess whether the research was conducted responsibly. Additionally, research funders and other stakeholders cannot evaluate how stakeholder views influenced study design or dissemination; this reduces accountability. Under-reportong of PPIE can also hinder evidence-based improvements in PPIE practice.

On an ethical level, under-reporting of PPIE can devalue the contributions of patients and public stakeholders, which may discourage future engagement. The quality and relevance of research can be difficult to determine when there are no visible records of PPIE. Inadequate reporting of any methodology also prevents reproducibility, which is a key theme in responsible research practice.

Overall, under-reporting of PPIE can weaken the transparency and integrity of the research process, and can also reduce its impact and reproducibility. Improved reporting through the use of standardised guidelines such as GRIPP2, can promote more inclusive, accountable and effective research practices.

Regardless of likely under-reporting of PPIE in Otolaryngology research, PPIE incorporation into trials appears to have remained low in recent years. For example, since the introduction of the GRIPP2 checklist, research has identified that reporting of PPIE in trials is low [36, 37]. Even in trials where patient involvement is expected, such as patient-focussed research, PPIE has been observed to be absent or poorly reported [38, 39]. It is thus likely that the limited number of Otolaryngology trials reporting on the use of PPIE identified by this systematic review is a true reflection of the limited uptake of PPIE in Otolaryngology research.

Given that PPIE is known to improve the quality and patient-centredness of research and allow PPIE contributors to feel empowered by sharing their lived experience [7, 40]the limited uptake of PPIE is concerning for the field of Otolaryngology. Otolaryngology research benefits from patient involvement as it involves the study of many interventions which would benefit from being designed in a patient focused manner, including interventions to aid communication and improve quality of life. It is therefore unsurprising that most of the articles utilising PPIE in this review were otology and audiology themed, a field which is ideal for PPIE due to the nature and context of diseases treated [19, 20].

Although PPIE has recognised benefits, low uptake of PPIE in research may be attributed to challenges associated with its use. For example, research has identified concerns amongst researchers regarding costs [41] and unfamiliarity surrounding PPIE incorporation [6]. Suggested strategies for increasing uptake include recognised successful approaches such as major funding bodies encouraging its use [1–4].

Where PPIE has been utilised in research, adherence to the GRIPP2 checklist has been found to be low. Of the 10 studies forming the final synthesis in this review, no studies directly referred to the GRIPP2 checklist. Adherence to the recommended domains in the GRIPP2 checklist was poor, especially regarding critical reflection on the use and influence of PPIE and its influence. These sections of the checklist were unaddressed. Of the studies used for data extraction, 90% (*n* = 9) included PPIE use in the methods section. Of these, 22% (*n* = 1) also mentioned PPIE use in the acknowledgements Sect. 10% (*n* = 1) of the 10 studies analysed mentioned PPIE use solely in the acknowledgements section. This led to challenges in conduction of this systematic review as all papers of interest required full-text screening in place of initial title and abstract screening to identify whether PPIE had been utilised. These findings are not limited to this review. Quality of reporting of PPIE according to the GRIPP2-SF checklist, and reference to this checklist, has been found to be poor in existing literature [36, 37].

### Suggestions for enhancing PPIE practice and policy

Identifying deficiencies in practice is not useful without recommendations to enable more robust implementation of PPIE in research. One recommended strategy is journals requiring authors to explicitly state the presence or lack of PPIE in submitted manuscripts [42]. The inclusion of PPIE in journals’ author guidelines and manuscript submission instructions may raise awareness of the importance of PPIE in responsible research. A further step may involve the recommendation of structured abstract sections explicitly addressing PPIE for relevant studies, or inclusion of the GRIPP2 adherence checklist as part of journal submission guidelines, for major research trials.

The training of peer reviewers and journal editors, sub-editors and assistant editors in the basic principles of responsible PPIE would also be useful, and would enable the potential benefits of PPIE, to be flagged to authors, by peer reviewers and editors.

As well as ensuring that PPIE use is documented in published manuscripts, the use of keywords (e.g. “PPIE,” “patient involvement” to improve discoverability is also recommended.

Recognition of a patient’s involvement in research serves as acknowledgment; PPIE contributors can be considered full stakeholders in a research team, highlighting the importance of responsible PPIE practice and engagement.

Nevertheless, it could be considered that addressing all items in the GRIPP2 checklist may sometimes be unnecessary, irrelevant, or impossible. To overcome this, this systematic review utilised the GRIPP2-SF instead of the GRIPP2-LF. The former is a checklist created for use in studies to assess the quality of reporting PPIE use in trials where PPIE is not the main research focus [22].

### Strengths and limitations

This systematic review is the first to assess use of PPIE in Otolaryngology randomised controlled trials that were published from 2018 to 2023, with the aim to identify reporting of PPIE in line with GRIPP2 checklist and assess impact on patients and clinical outcomes. We have identified ENT trials reporting on PPIE, trial study characteristics, trial adherence to the GRIPP2 checklist, and identified areas requiring future PPIE research.

However, this systematic review is not without its limitations. Due to a lack of information regarding PPIE use in study abstracts, a high number of full text manuscripts were reviewed by all authors, introducing potential for bias arising from large-scale manual screening errors, as well as from language and publication bias. However, reviewer agreement was very high at 99.4%, with only 2/346 cases requiring arbitration; these were resolved through independent re-review by the senior author. Kappa scores of inter-reviewer agreement were therefore not deemed to be required. Due to the large number of full text manuscripts that were reviewed and found not to include mention of PPIE, authors were not contacted for missing PPIE details as this was not deemed feasible, and potentially unreliable in the event of non-response.

It was not possible to determine the impact of PPIE on patients or clinical outcomes due to low quality reporting of PPIE, and difficulty in identifying the direct benefits of PPIE. As observed in studies identified by this review, PPIE delivery is often not conducted in a controlled manner which would allow for direct impact of PPIE on outcomes to be identified.

Additionally, it is likely that our estimate of the number of PPIE inclusive trials in ENT is likely an underestimation due to under-reporting of PPIE, and that PPIE input where reported may be under-represented. The discrepancy between planned and reported PPIE is recognised [35]and interpretation of what PPIE is may vary. For example, we were able to identify a study which confirmed the use of PPIE, but we did not deem it to be true PPIE in line with widely accepted definitions [43]. Thus, our interpretation of PPIE statements at face value, and not enquiring further by contacting authors, is a limitation of this systematic review.

## Conclusion

There remains a low volume of reported PPIE incorporation in Otolaryngology research, with very few Otolaryngology articles reporting on the utilisation of PPIE. Low quality reporting which is not in line with the GRIPP2, and poorly controlled PPIE use, mean that the impact of PPIE could not be determined. The lack of reporting of PPIE in study abstracts, overall lack of confirmation of PPIE use in articles, and poor adherence to the GRIPP2 are barriers to identifying the true nature and impact of PPIE utilisation in Otolaryngology research. Importantly, there is a need to continue raising awareness of the benefits of PPIE, to increase its implementation by researchers, particularly in Otolaryngology-associated fields other than otology and audiology. PPIE reporting should be made compulsory including in the abstract where relevant, and reporting in line with the GRIPP2 should be encouraged.

Future trials could incorporate PPIE in a more focused and structured way, to improve the ability to directly attribute outcomes to PPIE. Higher quality reporting will gradually lead to the development of a stronger PPIE evidence base that will facilitate more effective synthesis of PPIE studies. More effective synthesis of the PPIE evidence base allows for the identification of best practice, and contributes to research that is relevant, appropriate, and high-quality, which has the potential to generate benefit for all stakeholders.

## Data Availability

Primary data are available as supplemental files. This study was pre-registered on the PROSPERO database.
